# Efficient TALEN-mediated *myostatin* gene editing in goats

**DOI:** 10.1186/s12861-016-0126-9

**Published:** 2016-07-27

**Authors:** Baoli Yu, Rui Lu, Yuguo Yuan, Ting Zhang, Shaozheng Song, Zhengqiang Qi, Bin Shao, Mengmin Zhu, Fei Mi, Yong Cheng

**Affiliations:** College of Veterinary Medicine, Yangzhou University, No. 12 Wenhui Road, Yangzhou, 225009 Jiangsu Province People’s Republic of China

**Keywords:** *Myostatin*, TALENs, Mutational goat, Muscularity, Nuclear transfer

## Abstract

**Background:**

*Myostatin* (*MSTN*) encodes a negative regulator of skeletal muscle mass that might have applications for promoting muscle growth in livestock. In this study, we aimed to test whether targeted *MSTN* editing, mediated by transcription activator-like effector nucleases (TALENs), is a viable approach to create myostatin-modified goats (*Capra hircus*).

**Results:**

We obtained a pair of TALENs (MTAL-2) that could recognize and cut the targeted *MSTN* site in the goat genome. Fibroblasts from pedigreed goats were co-transfected with MTAL-2, and 272 monoclonal cell strains were confirmed to have mono- or bi-allelic mutations in *MSTN*. Ten cell strains with different genotypes were used as donor cells for somatic cell nuclear transfer, which produced three cloned kids (K179/MSTN^−/−^, K52-2/*MSTN*^+/−^, and K52-1/*MSTN*^+/+^).

**Conclusions:**

The results suggested that the MTAL-2 could disrupt *MSTN* efficiently in the goat genome. The mutated somatic cells could be used to produce *MSTN*-site mutated goats without developmental disruption. Thus, TALENs is an effective method for accurate genome editing to produce site-modified goats.

**Electronic supplementary material:**

The online version of this article (doi:10.1186/s12861-016-0126-9) contains supplementary material, which is available to authorized users.

## Background

Myostatin (encoded by the *MSTN* gene) is a member of the transforming growth factor family that acts as an important negative regulator of skeletal muscle growth [1, 2]. Mutations in *MSTN* can inactivate its expression and result in a nonfunctional protein, which has great potential to enhance muscle growth, leading to dramatic muscularity and a “double-muscling” phenomenon in many species, including cattle [3], mice [4], humans [5], and sheep [6]. Recent studies showed that inhibiting *MSTN* increases skeletal muscle mass, reduces fat mass, and inhibits diet-induced and genetic obesity [7–9], providing an opportunity to improve the production of meat in livestock. Several research institutions have attempted to produce *MSTN* knockout goats or sheep [9–13]. However, because of the low rate of conventional homologous recombination or zinc-finger nuclease (ZFN)-mediated knockout, there have been few reports of successful *MSTN* knockout goats [13].

Recently, the newly developed transcription activator-like effector nucleases (TALENs) and clustered regularly interspaced short palindromic repeats (CRISPR) techniques have emerged as powerful genetic tools to edit the genome precisely [14–17]. They represent a promising approach for targeted knockout at specific genomic loci, and have been used widely to perform precise genome editing in a wide range of organisms [15, 18–20]. Recent advances in the study of the CRISPR system have resulted in improved efficiency that allowed it to rapidly replace other genome editing platforms, including ZFNs and TALENs. Outstanding work has been done by one team of researchers using CRISPR/Cas9 to edit the goat genome. In that work, they produced MSTN mutated founders. These founders should be valuable for research into MSTN. At same time, some off-target modifications were detected in their founders [11]. However, as new breeds of goat for human consumption, we must avoid or reduce the risk of off-target mutations as much as possible. Some reports showed that no or few off-target mutations were detected in the animals created by TALENs [14, 21, 22]. The latest report also showed that the cloned cattle created by TALENs combined with SCNT are free of off-target events [23]. Thus, to reduce or avoid the induction of off-target mutations, we chose the TALEN approach for *MSTN* editing in goats. As far as we know, *MSTN*-knockout in goats using TALENs has not been reported.

In this paper, we tested whether direct *MSTN*-editing mediated by TALEN in goat cells is a viable approach to produce *MSTN* mutant goats. To accomplish this, we considered two objectives: (i) to design functional *MSTN*-modified TALENs; and (ii) to develop methods for TALEN-mediated *MSTN* modification in a cloned goat.

## Results

### Evaluation of TALENs efficiency

**Fig. 1:**
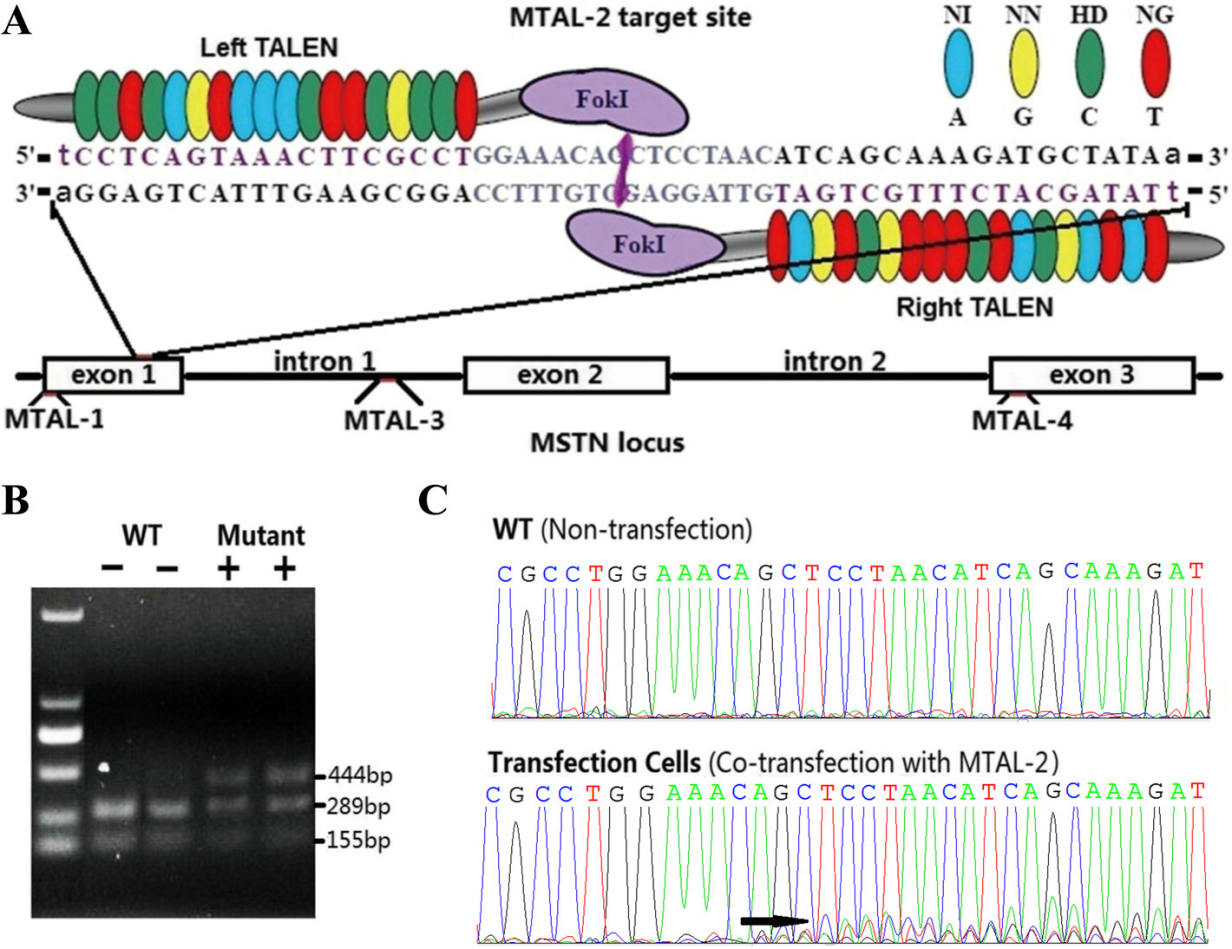
Design of the TALENs assay targeting the goat *MSTN* locus. **a** Schematic showing the DNA-binding sites and spacer sequence of MTAL-2 in *MSTN* exon 1. **b** Modification of *MSTN* in GFFCs when transfected with one pair of functional TALENs (MTAL-2). Surveyor nuclease cleavage of the unsorted cells’ PCR products produced an expected 444-bp band, while WT cells produced a 289-bp band and a 145-bp band. ‘- -’ means no additional band was observed; ‘+ +’ means the additional band was observed. **c** Mutation identified in mixed transfection cells. DNA fragments surrounding the TALEN target sites were analyzed by direct DNA sequencing. The sequencing ideogram shows a series of peaks after the targeting site

We designed and assembled four pair of TALENs (Additional file 1: Table S1) each to target the first exon, first intron and third exon of goat *MSTN*. Each TALEN pair was transfected into primary GFFCs (goat fetus fibroblast cells), and PCR was used to measure the efficiency of genome modification was measured at 72 h after transfection. Sequencing and restriction analyses of the amplicons revealed that one pair of TALENs (MTAL-2, targeting the first exon) resulted in detectable gene-editing activity. A schematic and the binding sequences of MTAL-2 are shown in Fig. 1a. Restriction analysis showed that the restriction enzyme could not completely cut the PCR amplicon from the mixed cells, when co-transfected with MTAL-2 expression plasmids (Fig. 1b). The sequencing ideograms of the cells transfected with MTAL-2 showed multiple peaks after the targeting site (Fig. 1c). The TA-cloning sequencing showed 7 of 36 TA-cloning were detected with mutation. The frequency of mutated alleles was estimated at about 19.4 %.

### Generation of MSTN-modified goat fetal fibroblasts

**Fig. 2:**
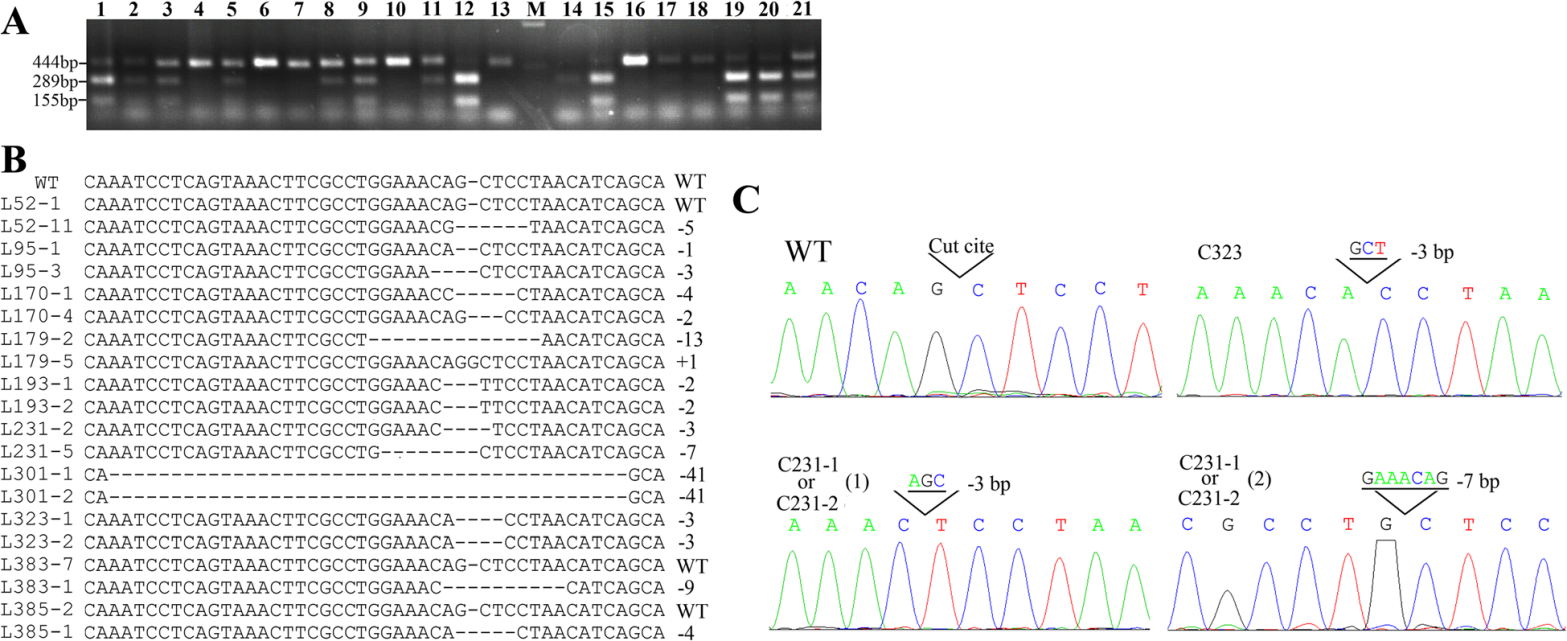
Sequence analysis of the *MSTN* locus in transfected cell strains. **a** Surveyor nuclease cleavage of the transfected cell strains. The amplicons from 21 cell strains were digested by restriction enzyme Alu I. Two bands were observed in wild-type cell strains, as shown in lanes 12, 14, and 15. Three bands were observed in mono-allelically modified cell strains or mixture cells, containing a 444-bp additional band, as shown in lanes 1–3, 5, 8, 9, 11, and 19–21. Only one 444-bp band was observed in bi-allelically modified cell strains, as shown in lanes 4, 6, 7, 10, 13, and 16–18. **b** Sequence variations at the cleavage site of 10 donor cell strains were aligned with the wild-type sequence. L52, L383, and L385 had mono-allelic mutations; L95, L170, L179, and L231 had bi-allelic mutations; and L193, L301, and L323 had homozygous mutations. ‘-’ means frameshift mutant; ‘+’ means insertion mutant. **c** Sequence analysis of the cloned fetuses. Three cloned fetuses (named C231-1, C231-2, and C323) were obtained from two recipients. C231-1 and C231-2, containing the same genotype, came from the bi-allelic mutant donor cell L231. C323 came from the homozygous mutant donor cell L323

Eleven days after transfection with MATL-2, 320 monoclonal cell strains were randomly isolated. An AluI restriction site was designed in the MTAL-2 cut site. Genotyping was performed by PCR amplification, AluI digestion, and TA-cloning sequencing. Additional bands were observed by PCR amplification of the target region in some of the cell strains (Fig. 2a), indicating that genomic modification had occurred in these cells. As shown in Fig. 2a, when the PCR products were digested with AluI, two bands were observed in wild-type cell strains, and a 444-bp additional band was observed in mono-allelically modified cell strains, while only one 444-bp band was observed in bi-allelically modified cell strains. The results indicated that 272 (85 %) cell strains were modified at the *MSTN* locus. Among these cells strains, 174 (64 %) were mono-allelically modified and 98 (36 %) were bi-allelically modified (Table 1). TA-cloning sequencing of all bi-allelically modified cell strains and 34 well-grown, mono-allelically modified cell strains showed that MTAL-2 induced small insertions or deletions in the target site, as shown in Fig. 2b. Three cell strains were identified as having homozygous mutations (Fig. 2b).

**Table 1:** Genotype analyses of MSTN-modified goat fetal fibroblasts

Genotype of monoclonal cells	Count	Percentage (%)	Homozygous mutations
Total	320		
Mono-allelic mutant	174	54.4	-
Bi-allelic mutant	98	30.6	3 (1 %)
Wild type	48	15	-

### Generation of *MSTN* mutant fetuses and goats

To produce *MSTN* mutant goats, 10 well-grown cell strains with different sexes and genotypes were selected for use as donor cells for somatic cell nuclear transfer (SCNT). As shown in Fig. 2b, three cell strains were mono-allelically modified, four were bi-allelically modified, and three were homozygously modified. Four hundred and three embryos were reconstructed and transferred into 29 synchronized recipients. Seven (24 %) recipients were confirmed as pregnant by transrectal ultrasonography 30 days after embryo transfer. Three fetuses were obtained from three pregnant recipients derived from donor cells of different genotypes (Fig. 2b). The fetal cell lines (named C231-1, C231-2 and C323) were isolated and expanded for use in recloning. Sequence analyses showed the all the fetuses had the same genotype as their donor cells. C231-1 and C231-2, derived from L231, had the same genotype and were modified biallelically. C323, derived from L323, was modified homozygously (Fig. 2b and c). The results suggested that TALEN-mediated *MSTN*-modified cells could support early embryonic development, and the mutations could be stably inherited.

**Fig. 3:**
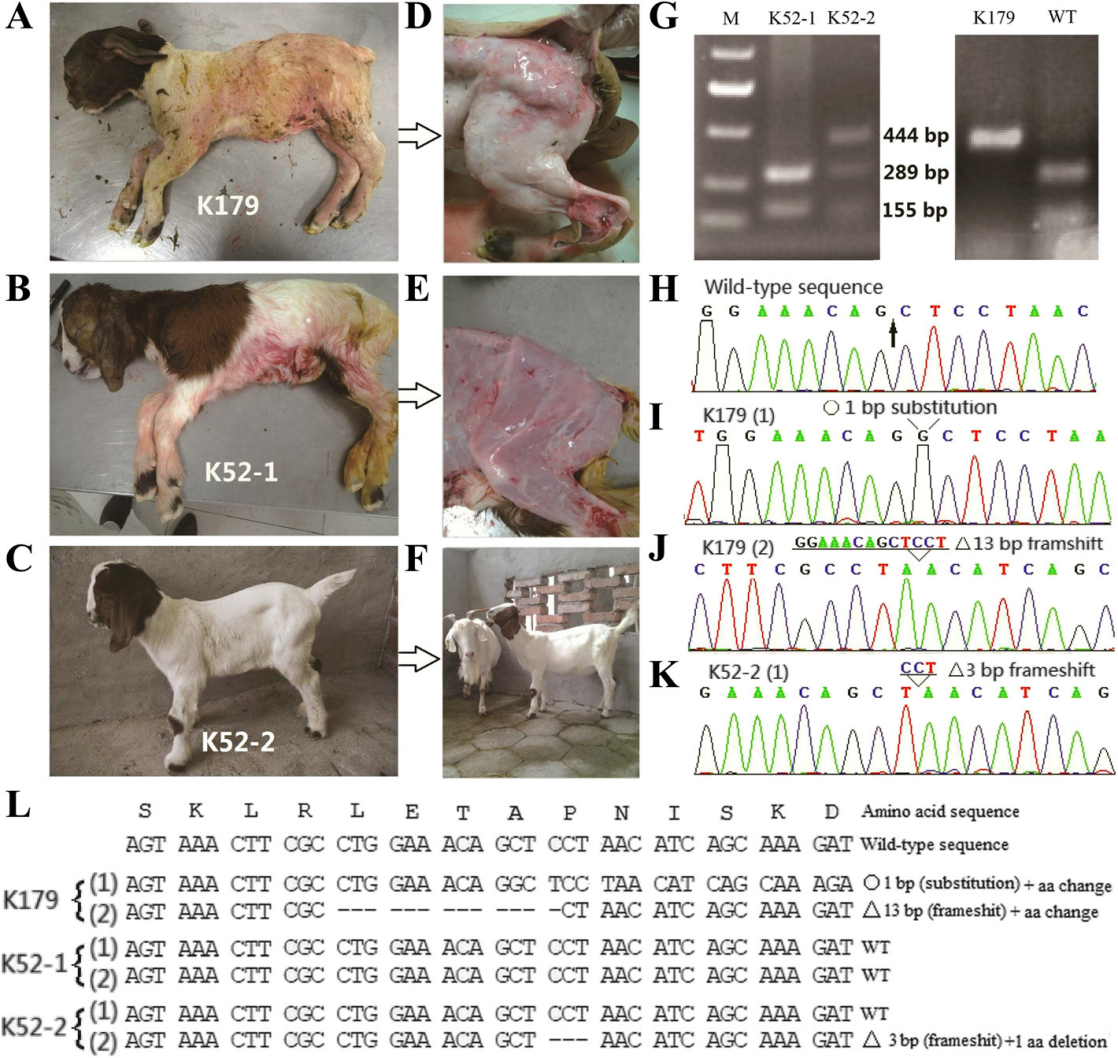
Phenotype and genotype analysis of cloned goats. **a**–**c** Photographs of the cloned goats at the first day after birth. **d**, **e** Phenotypes of K179/MSTN^−/−^ and K52-1/*MSTN* ^+/+^. The leg portion of the carcass. Pictures of the femoris muscles of skinned cloned goats are shown. **f** Picture of the live goat K52-2 at 4 months. **g** Mutation confirmation in the cloned goats. **h**–**k** Sequence analysis of the cloned goats. **l** Amino acid sequence analysis of the cloned goats

Two pregnant recipients carried their kids to term, delivering three female kids (named K179, K52-1, and K52-2; weighing 5.8 kg, 3.4 kg, and 4.5 kg, respectively) (Fig. 3a–c). However, K179 died because of a difficult labor and produced edema, K52-1 died 1 h after cesarean section, and K52-2 is currently alive and healthy (Fig. 3f). Restriction enzyme digestion showed K179 (derived from L179) was bi-allelically modified, and K52-2 (derived from L52) was mono-allelically modified, while K52-1 had no mutation (Fig. 3g). The results suggested that *MSTN*-modified cells produced by TALENs could support embryonic development, resulting in *MSTN*-modified goats.

### Characterization of cloned goats

**Fig. 4:**
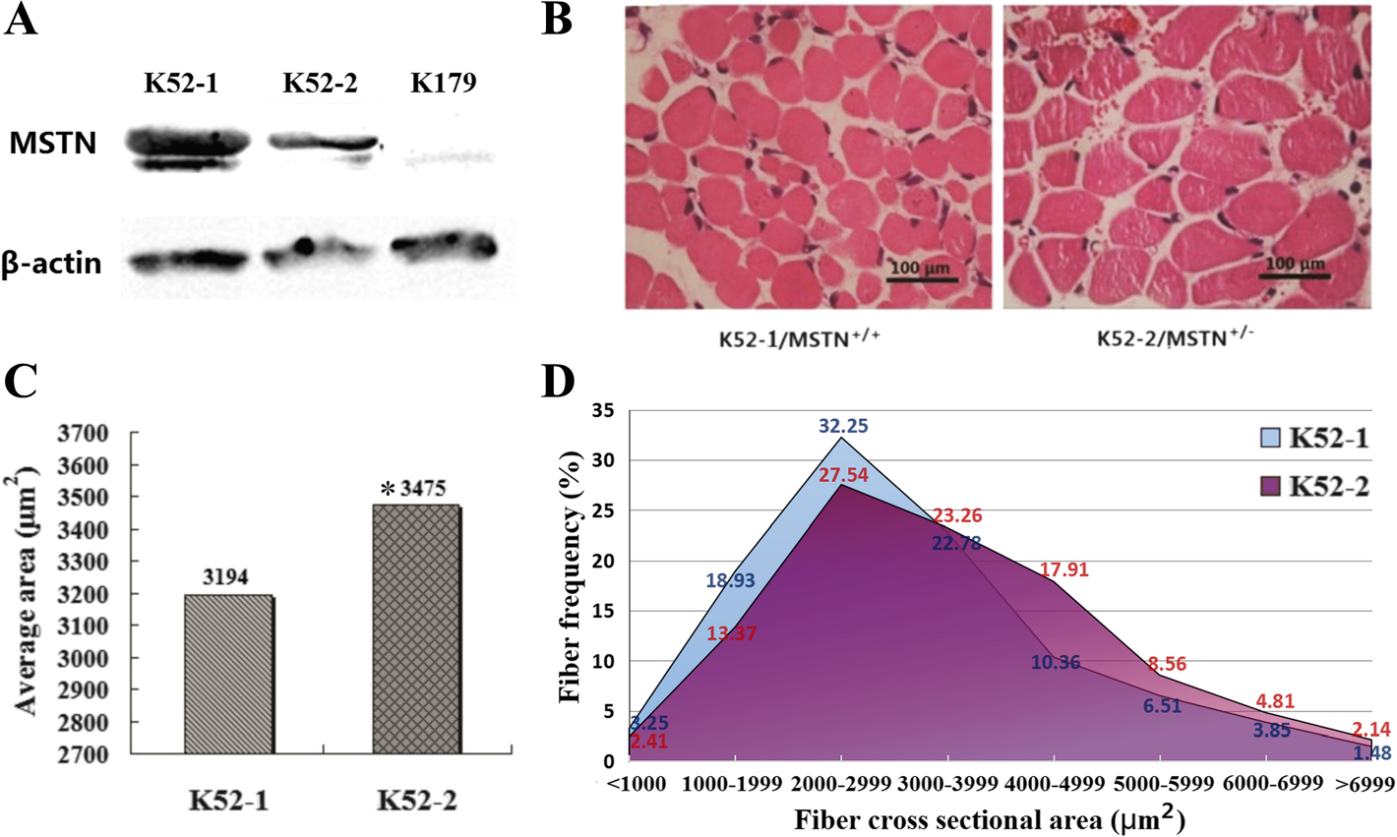
Myofibers and western blot analysis of *MSTN*-targeted goats. **a** Myostatin protein expression of K179, K52-1, and K52-2 was normalized to β-actin expression using western blot assays. **b** Muscle cross-sections of K52-2/*MSTN* ^+/−^ and K52-1/*MSTN* ^+/+^. Tissue sections were prepared from the fore limb tricep muscles that was dissected from the cloned goat 2 h after birth. **c**, **d** Mean average area and frequency distribution of the myofibers in a given cross-sectional area. In the K52-2/*MSTN* ^+/−^ and K52-1/*MSTN* ^+/+^ groups, 338–374 randomly selected myofibers was measured and used for analysis. *P value less than 0.05 considered as statistically significant. **d** The blue shaded area shows that more myofibers are in the 1000–3999 μm^2^ range in K52-1/*MSTN* ^+/+^; the red shaded area shows that more myofibers are in the 4000–6999 μm^2^ range in K52-2/*MSTN* ^+/−^

Sequence analyses of the cloned kids showed that a 1-bp insertion and a 13-bp deletion were present in the target site of the K179 kid (Fig. 3i and j), and a 3-bp deletion was detected in one allele of the K52-2 kid (Fig. 3k). The mutations in K179 were predicted to result in a null mutation because they occurred after the first 68 and 65 amino acids of the C-terminal region, respectively, resulting in the loss of 307 and 310 amino acids (Fig. 3l). The mutation in K52-2 resulted in a proline loss in the mature region of the protein (amino acid 70, Fig. 3l). Western blot analysis further demonstrated that myostatin was not expressed in K179 and K52-2 showed lower myostatin expression than K52-1 (Fig. 4a).

*MSTN* mutations in goats might lead to increased muscularity. Compared with the wild-type cloned kid K52-1/MSTN^+/+^, K179/MSTN^−/−^ showed a dramatic and widespread increase in skeletal muscle (Fig. 3d and e). The body weight of K179/MSTN^−/−^ was 1.7 times higher than K52-1/MSTN^+/+^, although this result could not be accurately estimated because of edema. Compared with the wild-type littermate K52-1/*MSTN*^+/+^, the body weight of K52-2/*MSTN*^+/−^ was increased by 32 % weight and the mean fiber area increased by 8.8 % (*P* < 0.05, Fig. 4b and c). Furthermore, K52-2/*MSTN*^+/−^ showed the same frequency distribution of myofibers (Fig. 4d) as was demonstrated in *MSTN*-knockout mice [24]. These results suggested that we generated a novel method to block *MSTN* expression in goats, which has potential to improve goat meat production.

### Off-target analysis

To assay the specificity of MTAL-2 induced DNA disruption, we employed the protocol described by Yong L et al. [25] to scan the genomic sequences in goats. The spacers between the two effector-binding elements in all the potential off-target cleavage sites were greater than 100-bp, which were too long to form Fok I dimerization. Thus, the PCR test that was performed in previous studies to confirm off-target sites was unnecessary [25–28]. In addition, the six most potential off-target cleavage sites were predicted (Additional file 2: Table S3) and analyzed by DNA sequencing. No evidence of off-target cleavage events could be identified. The information on the off-target loci and primer pairs used are listed in additional files (Additional file 2: Table S3 and Additional file 3: Table S4).

## Discussion

In this study, one pair of functional TALENs was identified and was used to generate bi- or mono-allelic mutant cells, which were used as donor cells for SCNT, resulting in bi- and mono-allelic-modified goats. These results showed that the combination TALEN-mediated genome editing with SCNT is a useful method to accomplish *MSTN* mutagenesis in goats.

In recent years, developments in ZFNs, TALENs, and CRISPR technologies have emerged as alternatives to classical breeding and transgenic methods to improve livestock. When deciding which technology to use in an experiment, it is very important to consider the final application of the mutated animals [17]. In this research, we attempted to create new breeds of goat to improve muscle yield, with the ultimate goal of approval for human consumption. To avoid or prevent significant risks of off-target mutations, we chose TALENs to achieve mutagenesis instead of CRISPR. Even though CRISPR has emerged recently as a potentially facile and efficient alternative to ZFNs and TALENs to induce targeted genetic alterations, CRISPR does not have better specificity than TALENs or ZFNs [17, 26]. Meanwhile previous studies have shown that low efficacy and minor off-target mutations may exist in TALEN-induced animals [22, 23, 28–31]. However, TALENs still provides a valuable option, thanks to its relatively high specificity [26]. Off-target analysis of the cloned goats revealed that no off-target sequences were detected. Additional studies will be required to evaluate the specificity of our functional TALENs.

Natural mutations in *MSTN* first exon [32], first intron [33], second exon [32], and third exon [34] have been found in animal populations, and all these mutations can lead to the double-muscle phenotype. Existing research shows that nucleotide variation among introns results in differential degrees of gene expression in eukaryotic cells [35]. In the present study, we tried to induce similar mutations in goat *MSTN* locus to knockout or reduce the expression of *MSTN*. The results indicated that MTAL-2, targeting for the first exon, could modify the *MSTN* locus. Most mutations induced by MTAL-2 were frameshifts in the target site, and the same mutation could be found at a high frequency in different cell strains. Cloned goats, derived from mutant cells with different sexes but containing the same mutation, can be used to produce homozygous mutant offspring by mating. In addition, the homozygous mutant cells can be used directly as donor cells for SCNT to produce homozygous animals.

SCNT is an important technology that has been used to clone certain livestock species [36]. Microinjection combined with TALEN mRNA is an efficient method that has been used to target *MSTN* in mice [37]. However, some reports stated that many of the resulting founder animals were chimeric, with multiple mutations [22, 27, 37]. The injection of TALENs into embryos is not as feasible in large animals as in small animals because large animals have longer gestation cycles and high recipient costs, which make it difficult to achieve large animal offspring with a single expected mutation [28]. The major advantage of SCNT over direct embryo injection with editor reagents is the predictable genotype of the offspring and the ability to generate clonal lines of edited animals [18]. Therefore, in this study, we chose expected genotype somatic cells modified by TALENs followed by SCNT to produce *MSTN* edited goats.

Three cloned goats derived from cells with the same genetic background were created. The *MSTN*-modified goats shared several hypermuscular features with *MSTN*-modified mice [37] or cattle. Our results showed that loss of myostatin did not disturb embryonic development, but led to increased body weight and mean fiber area.

## Conclusions

TALENs construct MATL-2 provided efficient *MSTN* gene disruption in the goat genome. The *MSTN*-modified cells could be used as donor cells for SCNT to produce *MSTN*-site mutated goats without disruption of embryonic development. Loss of myostatin protein resulted in muscular hypertrophy. These results represent an effective way to produce “double muscle” goat breeds.

## Methods

### Design and assembly of TALEN expression plasmids

The first exon, third exon, and its immediate flanking regions of the *Capra hircus MSTN* gene (NG_EF588034.1) were scanned for putative TALEN-binding pairs using TAL Effector Nucleotide Targeter 2.0 [38]. Four pairs of TALEN-binding pairs were chosen to construct TALEN expression plasmids using the FastTALETM TALEN Assembly Kit (Shanghai, China; Cat. No. 1802-030). The TALEN expression backbone contains a CMV promoter for expression in mammalian cells and a puromycin selection cassette for transient enrichment of transfected cells expressing TALENs.

### Animals

Yangtze River Delta white goats and Boer goats were kept in the Research Farm of Yangzhou University, China. All surgical procedures were performed in accordance with the Guiding Principles for the Care and Use of Laboratory Animals and were approved by the Institutional Animal Care and Use Committee of Yangzhou University.

### TALEN evaluation and edited cells

Goat fetal fibroblasts isolation and culture methods were the same as those described in a previous study [39]. 2 × 10^6^ cells were electroporated (Eppendorf multiporator) with 4 μg of the right-arm-TALEN encoding plasmid and 4 μg of the left-arm-TALEN encoding plasmid, under the following conditions: 400 V for 300 μs with one pulse. Empty backbone plasmid was used as a control. Puromycin (3 μg/mL) was added 24 h after transfection. Cells were collected at 72 hs after transfection. The targeted regions were PCR amplified (Primer sequences were shown in Additional file 4: Table S2) from genomic DNA and the amplicons were sequenced.

To obtain mutant cell strains, the functional TALEN expression plasmids were co-transfected into male or female goat fetal fibroblasts. Puromycin-resistant cells were cultured for another 7 days in normal medium, after which monoclonal cells could be observed. A cloning cylinder (C1059, Sigma) was then loaded around the single cell colony, and the cells were digested with trypsin. The monoclonal cell strains were then subcultured on 48-well culture plates. A portion of the cell culture was collected for cryopreservation, and the remaining cells were used for PCR screening to confirm the presence of the mutation. The PCR products were analyzed by restriction analysis and sequencing, before and after cloning into TA vectors.

### Nuclear transfer

The methods of animal treatment, oocyte collection, nuclear transfer, embryo transfer, and pregnancy status were as described in previous studies [39, 40]. Ten cell strains of different sexes were used as donor cells for SCNT. Several cell lines containing a desired mutant genotype were isolated from pregnant recipients and expanded to be used as donor cells for the next round of SCNT.

### Characterization of cloned kids

The cloned goats were weighed after birth, and their genomic DNA was extracted from the umbilical cord. To confirm the mutation, the PCR products of the cloned goats were analyzed by restriction analysis and sequencing before and after cloning into TA vectors. To observe the phenotype of the cloned goats, the dead goats were dissected and observed. The tricep muscles from the fore limbs in the cloned goats were isolated by a minimally invasive surgery at birth. Muscles were collected from dead goats and isolated from surviving goats by a minimal invasive procedure to make tissue sections and to extract proteins. The myofibers were subjected to straining and measured using the method reported in a previous study [24]. Muscle sections were photographed at 400× magnification. The muscle cross-sectional area was calculated using ImageJ software (National Institutes of Health, New York, USA). Data for myo-fibers were analyzed by a general linear model of regression using SPSS (General Linear Model, SPSS 11.0; SPSS, Inc., USA). Western blot analysis was conducted using a standard protocol. Antibodies for myostatin (Cat. No. ab98337), β-actin (Cat. No. ab8229), and goat polyclonal secondary antibody for rabbit IgG (Cat. No. ab97080) were purchased from the AbCam Company (AbCam, Inc., Cambridge, MA, USA).

### Off-target assay

To verify whether off-target mutations occurred in cloned goats, the target sequence of the active TALENs was searched in web software (http://blast.ncbi.nlm.nih.gov/Blast.cgi). The potential off-target sites were evaluated by sequencing.

## Abbreviations

GFFC, goat fetal fibroblast cell; MSTN, *myostatin*; SCNT, somatic cell nuclear transfer; TALEN, transcription activator–like effector nucleases

## Additional files

Additional file 1: Table S1. TALEN target sequences. (DOC 35 kb) Additional file 2: Table S3. Off-target sites assayed for MTAL-2. (DOC 83 kb) Additional file 3: Table S4. Primer pairs for PCR amplification of off-target regions for MTAL-2. (DOC 32 kb) Additional file 4: Table S2. Primers for PCR amplification of TALENs target regions. (DOC 29 kb)
