# Supplementary material for: Efficient TALEN-mediated myostatin gene editing in goats
Source: BMC Dev Biol. 2016 Jul 27;16:26. doi: 10.1186/s12861-016-0126-9 (PMC4962387; doi:10.1186/s12861-016-0126-9)
Supplement: Additional file 1: Table S1. — TALEN target sequences. (DOC 35 kb) [file 12861_2016_126_MOESM1_ESM.doc]

**Additional file 1:**

**Table S1** TALEN target sequences

| TALENs | TALE | TALE binding sequence | Spacer | Spacer Length (bp) | Localization |
| --- | --- | --- | --- | --- | --- |
| MTAL-1 | Left arm | TGTATTGATTTAAAAA | accatgcaaaaactg | 15 | exon 1 |
|  | Right arm | AAATATAAACAAAGATTTG |  |  |  |
| MTAL-2 | Left arm | CCTCAGTAAACTTCGCCT | ggaaacagctcctaac | 16 | exon 1 |
|  | Right arm | TATAGCATCTTTGCTGAT |  |  |  |
| MTAL-3 | Left arm | ATTTTGCTGTTATGAATAA | atgctacatattttt | 15 | intron 1 |
|  | Right arm | ATTAGCCTTTTAAAATGG |  |  |  |
| MTAL-4 | Left arm | TGATTGTGATGAGCACTC | cacagaatctcgat | 14 | exon 3 |
|  | Right arm | TAGAGGGTAACGACAG |  |  |  |
