# Supplementary material for: Efficient TALEN-mediated myostatin gene editing in goats
Source: BMC Dev Biol. 2016 Jul 27;16:26. doi: 10.1186/s12861-016-0126-9 (PMC4962387; doi:10.1186/s12861-016-0126-9)
Supplement: Additional file 2: Table S3. — Off-target sites assayed for MTAL-2. (DOC 83 kb) [file 12861_2016_126_MOESM2_ESM.doc]

**Additional file 3:**

**Table S3 Off-target sites assayed for MTAL-2**

|  | Left arm TALE-targeting sequence | | | | | | | | | | | | | | | | | | | Chr Locus | spacer | Chr Locus | Right arm TALE-targeting sequence | | | | | | | | | | | | | | | | | |
| --- | --- | --- | --- | --- | --- | --- | --- | --- | --- | --- | --- | --- | --- | --- | --- | --- | --- | --- | --- | --- | --- | --- | --- | --- | --- | --- | --- | --- | --- | --- | --- | --- | --- | --- | --- | --- | --- | --- | --- | --- |
| **MTAL-2 (Left arm TALE) spacer MTAL-2 (Right arm TALE)** | | | | | | | | | | | | | | | | | | | | | | | | | | | | | | | | | | | | | | | | |
|  | T | C | C | T | C | A | G | T | A | A | A | C | T | T | C | G | C | C | T |  |  |  | T | A | T | A | G | C | A | T | C | T | T | T | G | C | T | G | A | T |
| OTS1 | G | C | C | T | C | A | G | T | G | A | A | A | T | T | C | C | C | C | T | 10:86541984-86542002 | 697 | 10:86542680-86542663 | C | A | T | A | G | C | A | T | C | T | T | T | A | C | T | C | A | T |
| OTS2 | G | C | A | T | C | A | T | T | A | A | G | C | T | T | C | G | C | C | T | 24:3961039-3961057 | 573 | 24:3961611-3961594 | G | T | G | A | C | T | A | T | C | T | T | T | G | C | T | G | A | T |
| **MTAL-2 (Right arm TALE) spacer MTAL-2 (Right arm TALE)** | | | | | | | | | | | | | | | | | | | | | | | | | | | | | | | | | | | | | | | | |
|  | T | A | T | A | G | C | A | T | C | T | T | T | G | C | T | G | A | T |  | |  |  | T | A | T | A | G | C | A | T | C | T | T | T | G | C | T | G | A | T |
| OTS3 | T | C | A | A | G | C | A | T | C | T | T | T | T | C | T | G | A | C | 7:37288183-37288166 | | 128 | 7:37288056-37288073 | T | T | T | A | G | T | T | T | C | T | T | T | G | C | T | G | A | T |
| OTS4 | G | G | G | A | G | A | A | T | C | T | T | T | G | C | T | G | A | T | X:119165116-119165099 | | 469 | X:119164648-119164665 | T | T | C | A | G | A | A | T | C | T | T | T | G | C | T | G | A | T |
| OTS5 | A | T | T | G | G | C | A | T | A | T | T | T | G | C | T | G | A | T | 22:22804391-22804374 | | 602 | 22:22803790-22803807 | T | C | A | A | G | C | A | T | C | T | T | T | T | C | T | G | A | A |
| OTS6 | A | G | A | G | G | C | A | T | C | T | G | T | G | C | T | G | A | T | 13:1846691-1846674 | | 157 | 13:1846535-1846552 | C | A | G | T | A | C | A | T | C | T | T | T | G | C | T | G | A | A |
