# Supplementary material for: Efficient TALEN-mediated myostatin gene editing in goats
Source: BMC Dev Biol. 2016 Jul 27;16:26. doi: 10.1186/s12861-016-0126-9 (PMC4962387; doi:10.1186/s12861-016-0126-9)
Supplement: Additional file 3: Table S4. — Primer pairs for PCR amplification of off-target regions for MTAL-2. (DOC 32 kb) [file 12861_2016_126_MOESM3_ESM.doc]

**Additional file 4:**

**Table S4 Primer pairs for PCR amplification of off-target regions for MTAL-2**

| Primer | Sequence | Amplicons (bp) |
| --- | --- | --- |
| OTS1 For | ATCCTTACTGTCTTGTCT | 983 |
| OTS1 Rev | CACTCCTTAATATTTACC |
| OTS2 For | AGCAGGTGGTATCCGAGC | 698 |
| OTS2 Rev | TATGGTTTATTTTTGTCC |
| OTS3 For | AACATTAGGTTGCACAGC | 401 |
| OTS3 Rev | AATCATCCAAGGAGAAGA |
| OTS4 For | AGGTCACAAAGAGGCGAC | 972 |
| OTS4 Rev | AGGGAGATGAACCAGCAG |
| OTS5 For | ATCACATCCTGGGTCATA | 989 |
| OTS5 Rev | GCCTGTAGTTTTCTTTTT |
| OTS6 For | TAAATGAGGGATGGACAG | 523 |
| OTS6 Rev | AAAGAAGCAGAAGATGGG |
