# Supplementary material for: Efficient TALEN-mediated myostatin gene editing in goats
Source: BMC Dev Biol. 2016 Jul 27;16:26. doi: 10.1186/s12861-016-0126-9 (PMC4962387; doi:10.1186/s12861-016-0126-9)
Supplement: Additional file 4: Table S2. — Primers for PCR amplification of TALENs target regions. (DOC 29 kb) [file 12861_2016_126_MOESM4_ESM.doc]

**Additional file 2**

**Table S2. Primers for PCR amplification of TALENs target regions**

| Primer | Sequence | Amplicon (bp) |
| --- | --- | --- |
| MTAL-1 For | TATGCTGCTTGTTGCTGG | 322 |
| MTAL-1 Rev | GGTAATGACCGTTTCCGT |
| MTAL-2 For | TTGGCTTGGCGTTACTC | 444 |
| MTAL-2 Rev | TGACCGTTTCCGTCGTA |
| MTAL-3 For | TCTCTCTAATCATCATCA | 513 |
| MTAL-3 Rev | AGTATACCTTGTACCGTC |
| MTAL-4 For | TAACAGACACACCAAAAA | 347 |
| MTAL-4 Rev | ACAGCGATCTACTACCAT |
